# Highly Accurate Attitude Estimation of Unmanned Aerial Vehicle Payloads Using Low-Cost MEMS

**DOI:** 10.3390/mi16060632

**Published:** 2025-05-27

**Authors:** Xuyang Zhou, Long Chen, Changhao Sun, Wei Jia, Naixin Yi, Wei Sun

**Affiliations:** 1 School of Aerospace Science and Technology, Xidian University, Xi’an 710071, China; xuyangzhou@stu.xidian.edu.cn (X.Z.); 21131213336@stu.xidian.edu.cn (N.Y.); 2 CCTEG Xi’an Research Institute (Group) Co., Ltd., Xi’an 710077, China; chenlong@cctegxian.com; 3 Qian Xuesen Laboratory of Space Technology, China Academy of Space Technology, Beijing 100094, China; sunchanghao@spacechina.com; 4 365th Research Institute, Northwestern Polytechnical University, Xi’an 710072, China; jiawei@nwpu.edu.cn

**Keywords:** MEMS, error-state extended Kalman filter (ESKF), magnetic interference, orientation decoupling, attitude estimation, multifunctional payloads

## Abstract

Low-cost MEMS sensors are widely utilized in UAV platforms to address attitude estimation problems due to their compact size, low power consumption, and cost-effectiveness. Diverse UAV payloads pose new challenges for attitude estimation, such as magnetic interference environments and high dynamic environments. In this paper, we propose a hierarchical decoupled attitude estimation algorithm, termed HDAEA. Initially, a novel hierarchical decoupling approach is introduced for the attitude and angle representation of the direction cosine matrix, enabling the representation of angles in a new manner. This method reduces the data dimensionality and nonlinearity of observation equations. Furthermore, a magnetic interference identification algorithm is proposed to compute the magnetic interference intensity accurately and quantitatively. Combining the quantified errors of estimated state variables, an error model for magnetic interference and attitude angles in high-dynamic environments is constructed. Subsequently, the proposed error model is employed to calibrate the hierarchical decoupled angles using accelerometer and magnetometer measurements, effectively mitigating the impact of magnetic interference on the calculation of pitch angles and roll angles. Moreover, the integration of the proposed hierarchical decoupled attitude estimation algorithm with the error-state extended Kalman filter reduces system nonlinearity and minimizes linearization errors. Experimental results demonstrate that HDAEA exhibits significantly improved attitude estimation accuracy of UAV payloads.

## 1. Introduction

In recent years, unmanned aerial vehicles (UAVs) carrying payloads with different functions have been widely used in the fields of geographic mapping, firefighting, search and rescue, and military confrontation [1,2,3]. In the process of UAV mission execution, the accurate attitude of the payload platform is the basis for controlling the function of various payloads. With the development of microelectromechanical systems (MEMS), the accuracy and stability of the attitude measurement system based on low-cost MEMS design have been steadily improved, and it has gradually become the preferred solution for unmanned system attitude measurement [4,5,6].

A typical low-cost MEMS attitude measurement system consists of a three-axis gyroscope and a three-axis accelerometer. Combined with appropriate attitude estimation algorithms, these sensors can determine the roll angle and pitch angle attitude of the carrier [7,8]. At rest, the accelerometer can obtain a more accurate gravitational acceleration when the attitude measured by the sensor system is more reliable. However, during accelerated motion (especially vertical acceleration), accelerometers will not be able to provide an accurate orientation reference [9]. At the same time, the integration error of MEMS gyroscopes accumulates over time, which in turn leads to a degradation of the attitude estimation accuracy of the sensor system over time [10]. Nine-axis sensor systems, such as the attitude and heading reference system (AHRS), are formed by introducing a three-axis magnetometer and then combining it with an inertial sensor [11]. Researchers have developed several sensor fusion algorithms based on nine-axis MEMS sensors to achieve higher accuracy in 3D attitude estimation of roll angle, pitch angle, and azimuth for long-time carriers [12,13,14,15].

Obviously, the gravitational and geomagnetic fields can provide absolute observations of the three-dimensional attitude, and the algorithms mentioned above assume that the directions of the gravitational and geomagnetic fields are perpendicular to each other to obtain highly accurate attitude estimates. However, the real geomagnetic field is not as we assume, due to the magnetic inclination and magnetic field disturbances. When a non-orthogonal uniform magnetic field is used as a reference, the pitch and roll are not decoupled from the magnetic field [16,17,18]. In order to solve the problem of the effect of magnetic disturbances on the attitude angle, a commonly used attitude estimation algorithm is to allow the accelerometer and magnetometer to correct the attitude during the update process [19]. For the measured magnetic field vector, only the horizontal component is meaningful to observe, but its vertical component still affects the estimation of the pitch and roll. With the above method, the magnetometer measurements affect pitch and roll, even in an environment with a clean magnetic field [20]. The degree of distortion of the three attitude angles will be even more severe when magnetic field disturbances are present, which greatly reduces the engineering usefulness of the attitude estimation algorithm. There are two general research ideas for dealing with magnetic disturbances. The first one is to monitor the intensity of magnetic disturbances in real time and correlate it with the magnetometer observation noise [21,22]. Another idea is to recover the true geomagnetic information from the magnetometer data containing the disturbance. Reference [23] modeled a magnetometer and a six-axis IMU to solve a maximum likelihood problem, but it could not run in real time.

For nine-axis MEMS attitude estimation systems, the choice of models is divided into two main categories. The first category includes the classical Kalman filter (KF) applied to linear systems and its improvements for nonlinear systems. There is another class of attitude estimation algorithms represented by the complementary filter (CF) [24], which is used to update the attitude prediction of the gyroscope by controlling the proportional integration of the low-pass and high-pass filters combined with accelerometer and magnetometer measurements. In contrast, the gradient descent algorithm (GDA) [25] considers reducing the difference between the attitude estimation direction and the observed direction as an optimization problem. Through a series of optimization measures, the method estimates the direction of the uniform field and combines it with the gyroscope to obtain an optimal estimate. Compared with KFs, these two methods can obtain good real-time performance, but the accuracy is not as good as KFs. The extended Kalman filter (EKF) can act on nonlinear systems. Many scholars use EKF for attitude estimation in highly dynamic environments [26,27]. In order to solve the linearization error problem of EKF, some researchers proposed the iterated extended Kalman filter (IEKF) [28] to effectively reduce the nonlinear error, but at the same time, IEKF will introduce additional computational load. The environment we applied is a scenario with limited computing resources, so this iterative algorithm is not suitable. Reference [29] used adaptive attenuation root mean square UKF to estimate drill tool attitude. However, the unscented transformation process of UKF is more computationally intensive than the first-order Taylor expansion process of EKF, and the UKF algorithm also introduces two parameters to determine the selection of Sigma points, which brings great difficulty to parameter adjustment in practical engineering. There is also a group of researchers who have divided the 3D attitude estimation into two parts, using a two-layer Kalman filter to estimate different attitude angles [30,31,32]. Combined with the observability of the sensors, the estimation accuracy of the pitch and roll angles is improved. Regardless of the method chosen, these classical methods have a recognized weakness in that pitch and roll estimates are not, or not fully, decoupled from yaw estimates, leading to unpredictable attitude errors.

Typically, the absolute attitude of a UAV payload platform relative to the geo-referenced system is obtained by converting two sets of attitude angles, including the UAV AHRS measured attitude angle and the attitude angle of the payload stabilization platform. We mount a sensor system based on a low-cost MEMS design directly on the UAV payload platform. We constructed a hierarchical decoupled attitude estimation algorithm (HDAEA) in conjunction with the error-state extended Kalman filter (ESKF) [33], which improves the performance of the algorithm in highly dynamic and magnetically disturbed environments. The main contributions of this paper are as follows:

(1) A new framework for attitude estimation based on a low-cost MEMS sensor system is proposed. The attitude angle of the carrier relative to the geo-referenced system is directly estimated.
(2) By decoupling the attitude angle, the effect of high dynamic and magnetic field interference environment on the attitude angle estimation, especially on the azimuth angle estimation, is greatly reduced.

## 2. Materials

### 2.1. Data Acquisition Devices

**Figure 1:**
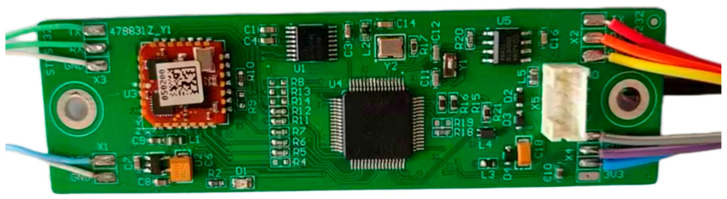
Physical map of the sensor assembly.

In this paper, a self-designed sensor module is used for the experiment. According to the overall design requirements, we chose STM32F103RCT6 (ST, Geneva, Switzerland) as the core processor. The inertial/magnetic sensor is the core device in this design. Considering factors such as sensor size, power consumption, operating temperature, measurement range, and accuracy, MTI-3-8A7G6T (Xsens, Enschede, The Netherlands) is selected as the sensor module of this design. The physical map of the sensor assembly is shown in Figure 1. The raw data output frequency of the sensor can reach up to 100 Hz, which meets the requirements of UAV scenarios. The specific parameters of the MTI-3-8A7G6T module are shown in Table 1.

### 2.2. Attitude Representation

**Figure 2:**
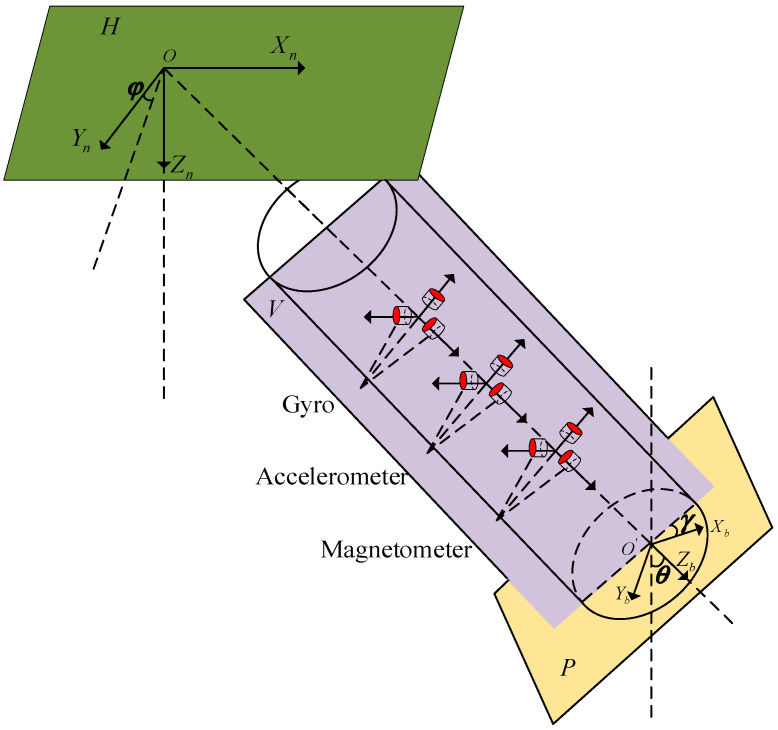
Installation diagram of sensors and coordinate system.

In order to realize the dynamic, high-precision, real-time attitude measurement of the UAV payload platform, it usually needs to include three sensors: an accelerometer, a gyroscope, and a magnetometer. As shown in Figure 2, the three-axis accelerometer, gyroscope, and magnetometer are installed in three mutually orthogonal directions of the UAV payload platform.

As shown in Figure 2, we select the right-handed rectangular coordinate system formed by the geographic coordinate system in the order of “north-east-down” as the navigation coordinate system (n-system) and express it with $OX_{n}Y_{n}Z_{n}$ to derive the calculation formula of attitude estimation. The payload coordinate system (b-system) is established with the three basic axes of the payload, which is represented by $O^{\prime }X_{b}Y_{b}Z_{b}$, where $Z_{b}$ coincides with the payload axis, $X_{b}$ and $Y_{b}$ are perpendicular to each other, and the plane formed by them is perpendicular to the payload axis.

The three basic parameters in the UAV payload platform are pitch angle, azimuth, and roll angle. Figure 2 depicts the Euler angle representation of the pose based on the previously described coordinate system. The pitch angle θ (θ∈(−90°,90°)) is the angle between the payload $Z_{b}$ axis and the normal line of the horizontal plane. The azimuth angle γ (γ∈(−180°,180°)) is the angle between the projection of the $X_{b}$ axis on the horizontal plane and the $X_{n}$ axis. The roll angle φ (φ∈(−180°,180°)) is the angle between the projection of the $Y_{b}$ axis on the $Y_{n}OZ_{n}$ plane and the $Y_{n}$ axis of the navigation system. The H-plane is the horizontal plane. The P-plane is the cross-section in the axial direction of the payload. The horizontal plane is rotated about the $X_{n}$ axis by an angle of φ which is the V-plane.

## 3. Methods

### 3.1. Hierarchical Decoupling of Attitude Angles

In general, Euler angles, quaternions, and direction cosine matrix (DCM) can represent the three-dimensional attitude angles of an object [34]. However, when the second order rotation angle is 90 degrees, Euler angles have a “gimbal lock” problem, which can lead to incorrect estimation of concurrency; the representation of quaternions is both compact and has no singularity. The disadvantage is that the expression information of each element to the angle is not clear, and the single angle cannot be decoupled. DCM uses nine quantities to describe the rotation of 3 degrees of freedom (3DOF), which is redundant, but the advantage is that each parameter is represented by the positive cosine value of the angle value, and the physical meaning is clear. The last line only contains the information of two angles and does not contain the information of azimuth. Combined with the Lie groups and Lie algebras, a new method of hierarchical decoupling of attitude and angle of orientation cosine matrix is proposed, which reduces the dimension of attitude representation and decouples its attitude angles, thereby obtaining a new way of representing the measurement of roll angle, pitch angle, and azimuth. The attitude angle to the rotation matrix should be rotated in an XYZ manner:

(1) Rbn=RφRθRγ=θcφc−γcφs+γsθsφcγsφs+γcθsφcθcφsγcφc+γsθsφs−γsφc+γcθsφs−θsγsθcγcθc

(2) R˙bn=Rbnωb∧

In the formula, the rotational angular velocity of the b-system with respect to the n-system is denoted as $\omega_{b}$, and (ωb)^ is its antisymmetric matrix.

The decoupled attitude is represented as:

(3) $$\left\{\begin{array}{l} r_{1}={\left[\begin{array}{ccc} -\theta_{s} & \gamma_{s}\theta_{c} & \gamma_{c}\theta_{c} \end{array}\right]}^{T}\\ r_{2}={\left[\begin{array}{cc} \theta_{c}\varphi_{c} & \theta_{c}\varphi_{s} \end{array}\right]}^{T} \end{array}\right.$$

$where\;g_{0}^{n}$ is the local acceleration vector, $g^{b}$ is the measurement value of the accelerometer in the payload coordinate system, and it can be known that the relationship between $r_{1}$, $g_{0}^{n}$, and $g^{b}$ is:

(4) gxbgybgzb=Rnb00−g0n⇒RbnT00−g0n=θsg0n−γsθcg0n−γcθcg0n=−θsγsθcγcθc−g0n=−g0nr1⇒gxbgybgzb=−g0nr1

As can be seen from (4), $r_{1}$ has a linear relationship with $g^{b}$, and $g_{0}^{n}$ is a constant factor. The observation equation becomes linear.

$m^{b}$ is the measurement value of the magnetometer. The sum of the first two elements of $m^{n}$ points to the geomagnetic north pole. The rotation matrix without azimuth information is expressed as D. The relationship between $m^{b}$ and $r_{2}$ can be obtained:

(5) $$D=\left[\begin{array}{ccc} \begin{array}{l} \theta_{c}\\ 0 \end{array} & \begin{array}{l} \theta_{s}\gamma_{s}\\ \gamma_{c} \end{array} & \begin{array}{l} \theta_{s}\gamma_{c}\\ -\gamma_{s} \end{array} \end{array}\right]$$

(6) $$\begin{array}{l} \left[\begin{array}{c} m_{x}^{n}\\ m_{y}^{n} \end{array}\right]=D\left[\begin{array}{c} m_{x}^{b}\\ m_{y}^{b}\\ m_{z}^{b} \end{array}\right]=\left[\begin{array}{c} m_{x}^{b}\theta_{c}+m_{y}^{b}\gamma_{s}\theta_{s}+m_{z}^{b}\gamma_{c}\theta_{s}\\ m_{y}^{b}\gamma_{c}-m_{z}^{b}\gamma_{s} \end{array}\right]\\ \Rightarrow m_{r_{2}}=\left[\begin{array}{c} m_{x}^{n}\\ m_{y}^{n} \end{array}\right]\cdot {\left(\sqrt{{\left(m_{y}^{n}\right)}^{2}+{\left(m_{y}^{n}\right)}^{2}}\right)}^{-1}\cdot \theta_{c}=r_{2} \end{array}$$

### 3.2. The First Layer Attitude Estimation Algorithm

**Figure 3:**
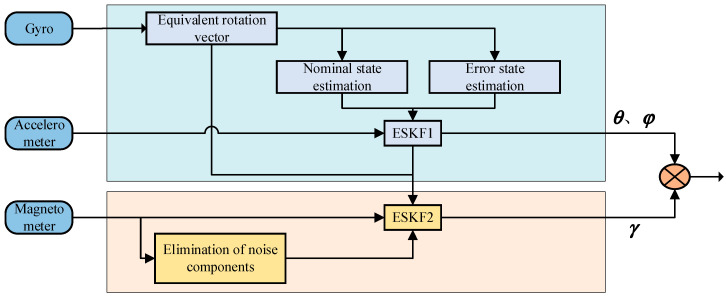
The overall block diagram of the algorithm.

The attitude angular error and gyro bias are used as state estimates for Kalman filtering. The error state dynamics equation and the error measurement equation are derived. Similar to [34], the process is reasonably simplified here. The block diagram of the hierarchical decoupled attitude estimation algorithm is shown in Figure 3.

The last row r of the attitude rotation matrix R from the payload coordinate system (b-system) to the navigation coordinate system (n-system) is chosen as the state variable expressing the pitch and the roll angles. In attitude estimation, the prediction equation is essentially a process of integrating the output value of the gyroscope, so the zero bias of the gyroscope can cause a large cumulative error. For this reason, the zero bias of the gyroscope is also treated as a state variable estimation.

The nominal state vector of the first layer is:

(7) $$x_{1}={\left[\begin{array}{cc} r_{1} & b_{g} \end{array}\right]}^{T}$$

where $r_{1}={\left[{-\theta_{s}\gamma }_{s}\theta_{c}\gamma_{c}\theta_{c}\right]}^{T}$.

From the above definition, let Φkb be the equivalent rotation vector in the time period $\left[k-1,k\right]$. Hence, the prediction equation can be written as:

(8) r¯1,k=r^1,k−1−(Φkb)∧r^1,k−1b¯g,k=b^g,k−1

The main principle of ESKF [35] is to treat a combination of the nominal state R and the error state matrix ϕ as the actual state $R_{t}$ (the error rotation matrix in Lie algebra is denoted as δϕ). It should be noted that the elements in R must satisfy the orthogonality constraint. In the code operation, the norm of the elements in the last row, which is estimated in the first layer, must be 1. From the differential equation $R_{t}=RExp\left(\delta \phi \right)$ of the rotation matrix, the relationship between the true state $r_{t}$ of the attitude angle and the nominal state r can be derived as follows:

(9) $$r_{t}=Exp{\left(\delta \phi \right)}^{T}r$$

where $Exp\left(\delta \phi \right)$ is the exponential mapping of the error state to $SO\left(3\right)$ [34].

The relationship between the real gyroscope bias and the error gyroscope bias is:

(10) $$b_{gt}=b_{g}+\delta b_{g}$$

The steps to update the error state are as follows:

(11) $$\left\{\begin{array}{l} K_{1,k}={\bar{P}}_{1,k}H_{1,k}^{T}{\left(H_{1,k}{\bar{P}}_{1,k}H_{1,k}^{T}+R_{1,k}\right)}^{-1}\\ \delta \hat{x}=\delta {\bar{x}}_{k}+K_{1,k}(a_{m,k}-{\bar{r}}_{1,k})\\ {\hat{P}}_{1,k}=(I_{6}-K_{1,k}H_{1,k}){\bar{P}}_{1,k} \end{array}\right.$$

According to (9) and (10), the nominal state is updated by the following formula:

(12) r^1,k=Exp(δr^k)r¯1,kTb^g,k=b¯g,k+δb^g,k

### 3.3. The Second Layer Attitude Estimation Algorithm

**Figure 4:**
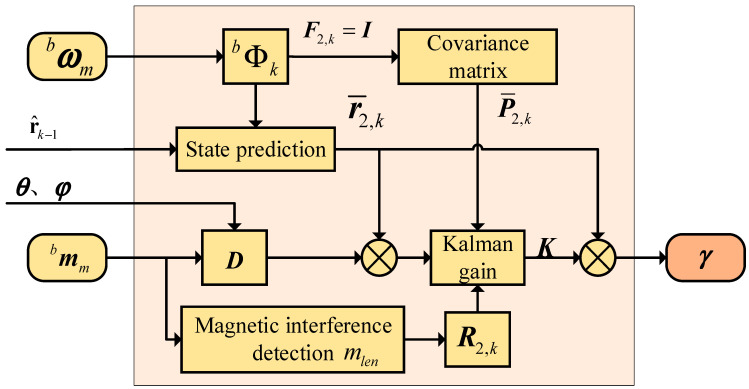
The second layer filtering flow chart.

In single-layer filtering algorithms, it is often necessary to compensate for external acceleration to minimize the effect of accelerometer measurement errors on azimuth estimation accuracy in highly dynamic environments [36]. The algorithm designed in this paper uses gyroscopes and magnetometers for azimuth estimation in the second layer of the filtering structure, avoiding the interference of accelerometer errors. We construct a figure of merit function (MF) characterizing the intensity of the magnetic interference and use the figure of merit function to compensate the observation matrix in the filter structure, which greatly improves the effect of the magnetic interference on the accuracy of azimuth estimation. The flow chart of the second layer filtering is shown in Figure 4.

The module magnetic field dip angle is the angle between the three-axis magnetometer composite magnetic vector of the module and the horizontal plane of the earth. In the actual calculation, it is indirectly calculated by the angle between the magnetic vector and the gravitational acceleration vector [6]. The calculation formula is as follows:

(13) $$dip=\left(\frac{\pi }{2}\right)-\arccos({\hat{g}}^{b}\cdot m_{m}^{b})$$

It should be noted that only when the module is in a pure geomagnetic field environment, the module magnetic field dip angle equal to the local magnetic dip angle. When there is external magnetic interference, the dip will change (may increase or decrease), and the direction of its angle change is uncertain. Here, we use the difference between the dip and the local magnetic dip to measure the intensity of the magnetic interference, which is $\left|{dip}_{ref}-dip\right|$. The occurrence of magnetic interference is not necessarily reflected in the change of dip. The magnetometer modulus value is also often used to measure the intensity of magnetic interference. Generally speaking, the stronger the magnetic interference, the larger the modulus value.

In view of the fact that magnetic interference is very complicated, the magnetic field inclination of the module or the magnetometer alone cannot measure the magnetic interference intensity. In order to measure the magnetic interference intensity more accurately, dip and $\left\|m_{m}^{b}\right\|$ are used to evaluate the magnetic interference intensity. The merit function is as follows:

(14) $$m_{len}=\log\left(\left\|m_{m}^{b}\right\|\sin\left(\left|dip-dip_{ref}\right|\right)\right)$$

In the above formula, $m_{len}$ is the magnitude of the magnetic interference. In order to allow the smaller magnetic interference to be detected in time, the logarithmic function is taken for the product of the two. To reduce the influence of the magnetic interference on the azimuth information, the intensity of the magnetic interference is correlated with the observation error matrix $R_{2,k}$ and the specific formula is as follows:

(15) $$R_{2,k}=\left(m_{len}\sigma_{d}^{2}+\sigma_{m}^{2}\right)I_{2}$$

where $\sigma_{m}$ is the standard deviation of the magnetometer noise and $\sigma_{d}$ is the basic standard deviation of the magnetic interference.

The state vector of the second layer is $x_{2}=r_{2}$, where $r_{2}={\left[\theta_{c}\varphi_{c}\theta_{c}\varphi_{s}\right]}^{T}$. We can easily know that the prediction equation of the orientation information is:

(16) x¯2,k=x^2,k−1+R12bnR13bnR22bnR23bnk−1Φz,k−Φy,k

(17) $${\bar{P}}_{2,k}=I_{2}{\hat{P}}_{2,k}I_{2}^{T}+Q_{2,k}$$

In the above formula, R12bn, R13bn, R22bn, and R23bn are calculated from ${\hat{r}}_{k-1}$ and ${\hat{x}}_{2,k-1}$.

The calibration process needs to introduce $x_{2}$ magnetometer to correct the heading information. The magnetometer data cannot directly correct the state, and it needs to be converted to the same dimension as the state $x_{2}$. The conversion value $m_{r_{2}}$ measured by the magnetometer can be obtained from (5).

(18) $$\left\{\begin{array}{l} K_{2,k}={\bar{P}}_{2,k}{\left({\bar{P}}_{2,k}+R_{2,k}\right)}^{-1}\\ {\hat{x}}_{k}={\bar{x}}_{k}+K_{2,k}(m_{r_{2}}-{\bar{x}}_{2,k})\\ {\hat{P}}_{2,k}=(I_{2}-K_{2,k}){\bar{P}}_{2,k} \end{array}\right.$$

where the observation noise matrix $R_{2,k}$ changes with the intensity of the magnetic interference. To sum up, the algorithm is shown in Algorithm 1.
**Algorithm 1:** Hierarchical Decoupled Attitude Estimation Algorithm**Input**: Triaxial angular velocity $\omega_{m},$ acceleration $a_{m}$, Magnetic field strength $m_{m}$.1. (θ,φ,γ) ← init_pose $\left(a_{m,0},m_{m,0}\right)$;2. Initialize: ${\hat{x}}_{1,0},\delta {\hat{x}}_{1,0},P_{1,0},Q_{1},R_{1},{\hat{x}}_{2,0},P_{2,0},Q_{2},R_{2},{dep}_{ref}$;**while** k>2(k=1,2,...,∝) **do:**  3. Construct the equivalent rotation vector $\Phi_{k}$ in Δt time;**The first layer of pose estimation(**θ,γ**)**  4. nominal_state prediction: ${\bar{x}}_{1,k}$;  5. error_state and covariance prediction: $\delta {\bar{x}}_{1,k}$, ${\bar{P}}_{1,k}$;  6. Compute the observation matrix: $H_{1,k}$;  7. error_state update: $\delta {\hat{x}}_{1,k}$;  8. error_state injection and reset: ${\hat{x}}_{1,k}={\bar{x}}_{1,k}⊕\delta {\hat{x}}_{1,k}$, $\delta \phi_{k}=0$, ${\hat{P}}_{1,k}=I_{6}{\hat{P}}_{1,k}I_{6}^{T}$;  9. (θ,φ) ⟵solve_angle(${\hat{x}}_{1,k}$);**The second layer of pose estimation(**φ**)**  10. State 2 prediction: ${\bar{x}}_{2,k}$;  11. Calculate magnetic interference: $m_{len}$;  12. Compute the observation error matrix: $R_{2,k}$;  13. state 2 update: ${\hat{x}}_{2,k}$;  14. $\left(\gamma \right)$ ← solve_angle(${\hat{x}}_{2,k}$);  15. **k = k + 1;****end while****Output →** Bit attitude (pitch *θ*, roll *φ*, azimuth *γ*)

## 4. Results and Discussion

In this section, we compare the proposed algorithm with EKF [26], ESKF [30], DEKF [31], and DOEA [32] to evaluate its accuracy in attitude estimation and efficiency under different experiments. In this paper, a self-designed sensor module is used for the experiment. See Section II for details on hardware design. To fairly evaluate and clearly observe the influence of magnetic disturbances, there are no preconditioning mechanisms utilized for the accelerometer and magnetometer in all algorithms during these experiments. In order to verify the accuracy of the algorithm, this experiment chose the attitude angle of the MTI-G-710 (Xsens, Enschede, The Netherlands) module as the reference. The algorithm parameters of this experiment are shown in Table 2. The algorithm was developed and tested on a PC but ultimately runs on a single-board Raspberry Pi 5.

A total of two sets of experiments are set up. In Experiment A, we aim to investigate the effect of magnetic field interference on the attitude estimation of the sensor system. During the experiment, the sensor module is fixed on a stable platform for the experiment in order to avoid as much as possible the interference from external acceleration. In Experiment B, the sensor system and monocular visual payload are mounted on the payload platform of the fixed-wing UAV ASN-216 (ASN, Xi’an, China), and the sensor system has been subject to external acceleration interference throughout the experiment. In the first half of experiment B, the UAV visual payload is controlled to be stationary, with respect to the UAV, when the sensor system is not affected by the servo motors of the payload platform. In the second half of experiment B, we activate the UAV payload platform servo motor to control the visual payload to track the ground target, at which time the sensor system is subject to changing magnetic field interference.

### 4.1. Experiment A

As mentioned above, using the new method of attitude angle decoupling, the algorithm HDAEA proposed in this article can completely eliminate the influence of magnetic interference on the roll angle and pitch angle when calculating the attitude. To this end, we place the module on a stabilizing platform, slowly rotate the module, and then allow the module to return to its initial state and remain stationary. While the module remains stationary, we use a magnet to move closer to the sensor module and then slowly move away. We ran the EKF algorithm, the ESKF algorithm, the DEKF algorithm, the DOEA algorithm, and the HDAEA algorithm proposed in this article, respectively, and then observed the degree of influence of the attitude angle.

**Figure 5:**
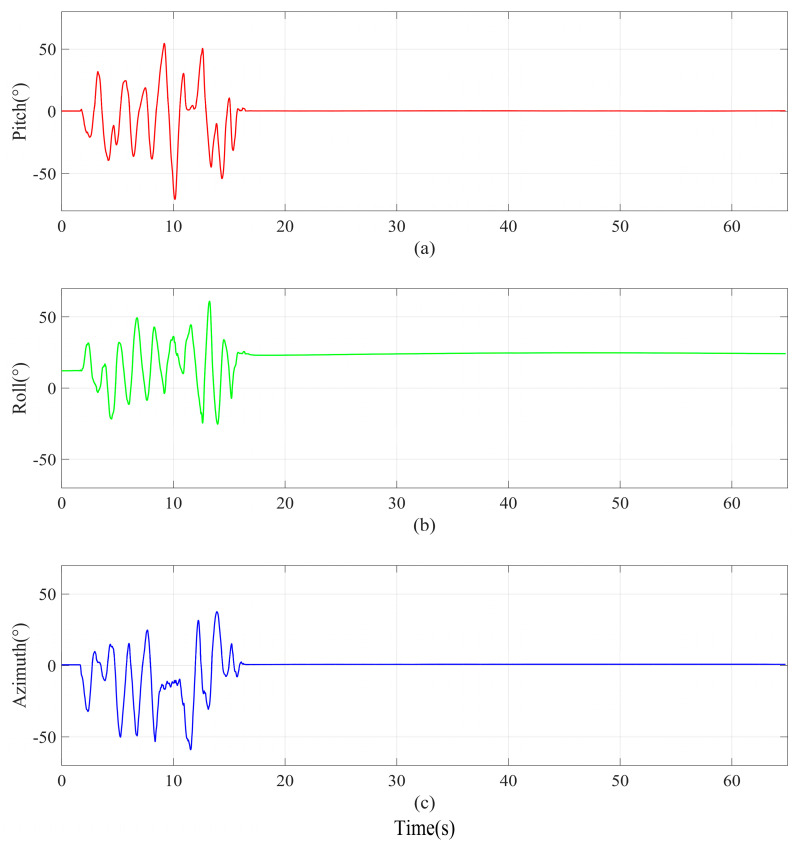
Attitude angle reference benchmark of experiment A. (**a**) Pitch. (**b**) Roll. (**c**) Azimuth.

As shown in Figure 5, the movement process of the module can be roughly divided into two periods. In the first period of 0~18 s, the module started to rotate slowly from rest, and in the second period of 18~64.9 s, the module was in a stationary state; then, it slowly approached the module with a magnet and finally moved away from it.

**Figure 6:**
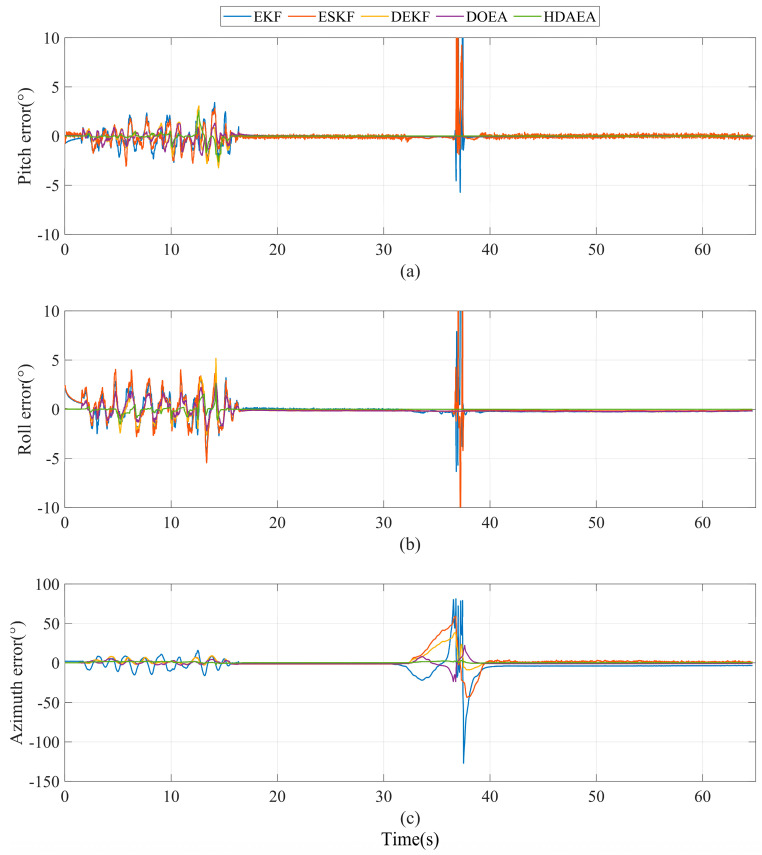
Comparison of attitude errors of five methods in experiment A. (**a**) Pitch error. (**b**) Roll error. (**c**) Azimuth error.

As shown in Figure 6, the EKF algorithm and the ESKF algorithm have no resistance to magnetic interference, and when the magnetic interference occurs, not only the azimuth, but also the pitch and roll angle are also affected to varying degrees because it allows the magnetometer and the accelerometer to correct the attitude at the same time. However, the pitch angle and roll angle calculated by the DEKF algorithm, the DOEA algorithm, and the algorithm proposed in this article are hardly affected by magnetic interference. Only the azimuth calculated by the algorithm proposed in this article is hardly affected by magnetic interference. In the static, pure magnetic interference environment, our algorithm achieves the highest accuracy.

The data statistics of the five algorithms are shown in Table 3. Compared with EKF, ESKF, DEKF, and DOEA, the algorithm proposed in this article has improved indicators, especially in azimuth, which has the largest improvement. The algorithm can stabilize the azimuth error within a reliable range. Experiments show that the proposed hierarchical attitude decoupling method can suppress the influence of magnetometer interference on azimuth.

**Figure 7:**
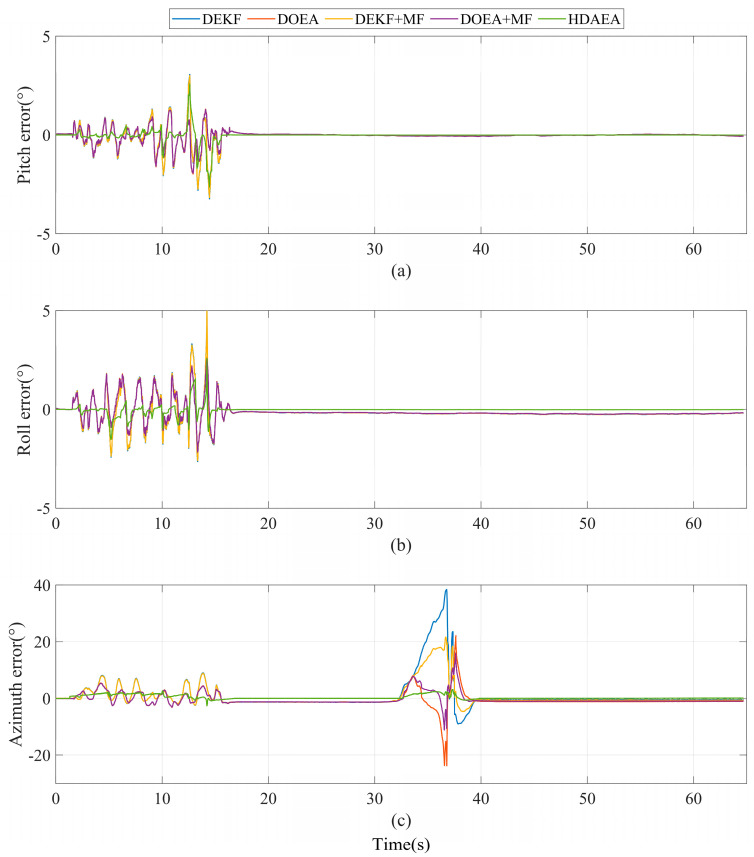
Comparison of attitude errors in the experiment to verify the validity of the merit function. (**a**) Pitch error. (**b**) Roll error. (**c**) Azimuth error.

In order to verify the validity of the merit function (MF) characterizing the intensity of the magnetic interference proposed in this paper, we use the figure of merit function to improve the second layer filter of the comparison methods DEKF and DOEA. In the same test environment, DEKF, EOEA, DEKF + MF, DOEA + MF, and HDAEA were used to estimate the attitude of the same set of sensor measurements. In the static, pure magnetic interference environment, both DEKF and DOEA algorithms optimized using the merit function have significantly improved azimuthal attitude estimation accuracy, as shown in Figure 7. However, our algorithm, HDAEA, still exhibits the highest accuracy. The data statistics are shown in Table 4.

### 4.2. Experiment B

**Figure 8:**
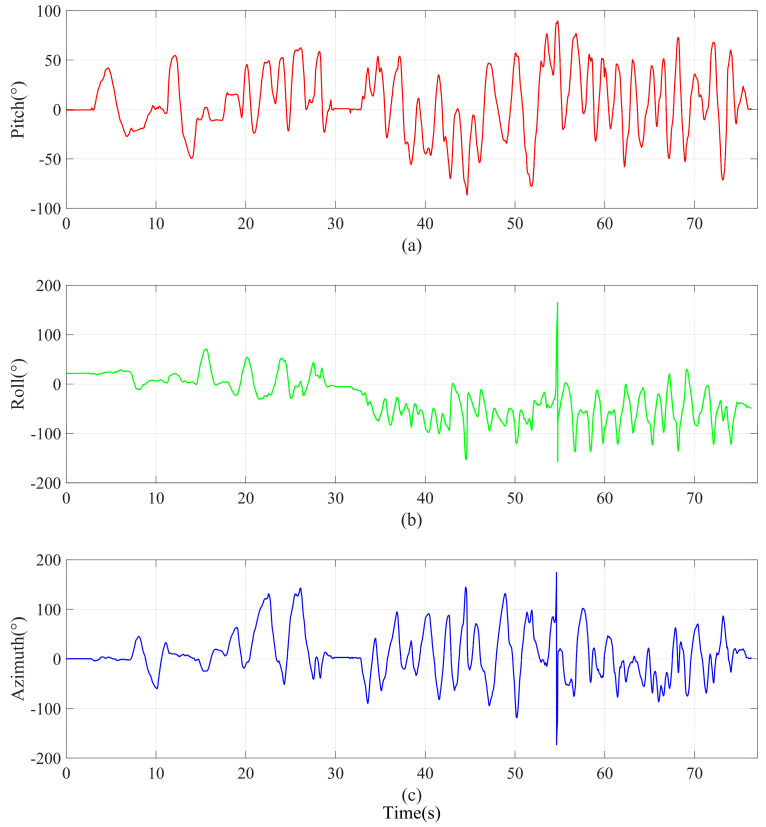
Attitude angle reference benchmark of experiment B. (**a**) Pitch. (**b**) Roll. (**c**) Azimuth.

In order to further evaluate and clearly observe the effects of external acceleration and magnetic field disturbances on UAV payload attitude estimation. We mounted the sensor module on a fixed-wing UAV for flight testing. We record the raw data output by the sensor and the attitude angle of the MTI-G-710 module. As shown in Figure 8, the attitude angle of the payload covered the full measurement range: the pitch angle θ (θ∈(−90°,90°)), the azimuth angle γ (γ∈(−180°,180°)), and the roll angle φ (φ∈(−180°,180°)). The movement process of the module can be roughly divided into two periods. In the first period, from 0 to 30 s, the attitude of the module changes slowly with the flight of the UAV. In the second period, from 30 to 76.4 s, the payload requires larger attitude adjustments during the tracking of the ground target by the UAV. We ran the EKF algorithm, the ESKF algorithm, the DEKF algorithm, the DOEA algorithm, and the HDAEA algorithm proposed in this article, respectively, and then observed the degree of influence of the attitude angle.

**Figure 9:**
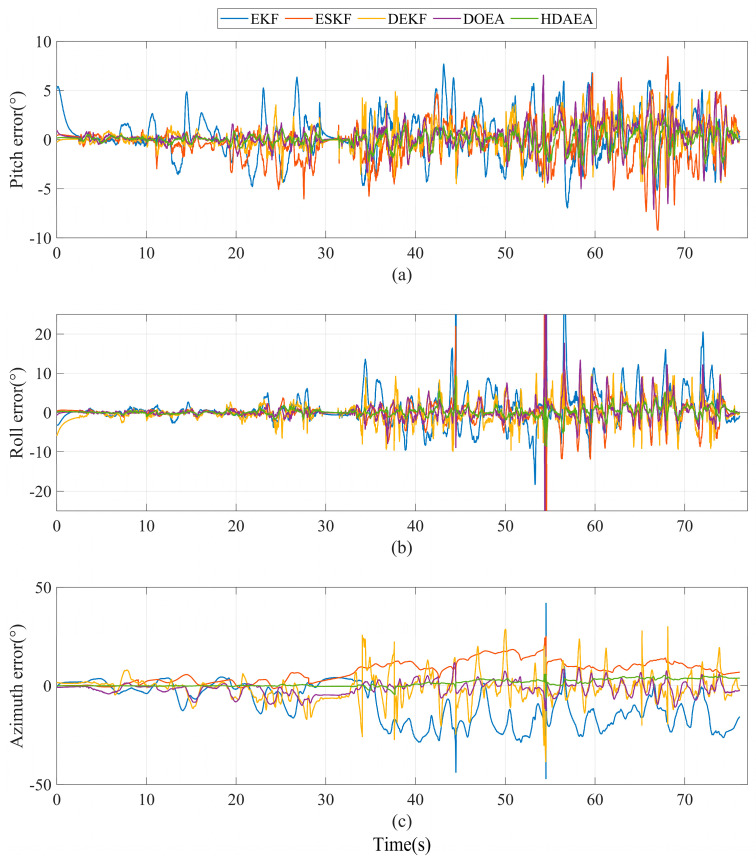
Comparison of attitude errors of five methods in experiment B. (**a**) Pitch error. (**b**) Roll error. (**c**) Azimuth error.

Assuming that the attitude movement range is large, if the attitude calculation result and the reference benchmark output by the module are directly displayed together, we cannot see the slight change trend. As shown in Figure 9, in order to show the details more clearly, here we compare the attitude angle calculated by the five algorithms with the reference datum output by the module.

In the first stage of the movement, we analyzed the data curves of the pitch and roll angle in Figure 9 and obtained that the angle errors solved by the DEKF algorithm and the DOEA algorithm were kept within ±7°, and the angular errors also remained within ±18° in the second segment of the movement. The angle errors solved by the algorithm in this article were kept within ±3° in the first segment of the movement, and the angular errors also remained within ±10° in the second segment of the movement. However, the angles obtained by the EKF algorithm and the ESKF algorithm are quite different from the reference angles. For the azimuth, the EKF algorithm, the ESKF algorithm, the DEKF algorithm, and the DOEA algorithm can still maintain a small error in the first 30 s, but the azimuth error is wildly inaccurate after magnetic interference, while for the algorithm HDAEA proposed in this article, the error compared to the reference angle remains within ±3° during the first segment of the movement range, and the error is within ±10° in the second motion. The data statistics of the five algorithms are shown in Table 5. Compared with EKF, ESKF, DEKF, and DOEA, the algorithm proposed in this article has improved indicators. In particular, the azimuth has the largest improvement, which can stabilize the azimuth error within a rational range.

## 5. Conclusions

The algorithm in this article solves the problem of the attitude estimation algorithm being prone to diverge, and the attitude angle is easily affected by magnetic interference due to the large nonlinear error in the high-dynamic scene. The algorithm decouples the rotation matrix into two layers to obtain a new attitude angle representation and derives a linear relationship between the decoupled angle of the first layer and the accelerometer measurements. Initially, the linearized filtered observation equation is established, which further reduces the external magnetic field interference that the magnetometer will inevitably encounter. In order to measure the magnetic field interference intensity more accurately, we designed a new magnetic interference identification method to quantify the magnetic interference intensity accurately. Then, we combine its measurement results with the state estimation error in the second layer filtering model to construct an error-based state estimation model. Finally, we construct a hierarchical decoupled attitude estimation model based on the error state, which improves the performance of the algorithm in high-dynamic and magnetic interference environments.

Based on the research results in this paper, related studies such as geo-localization of UAV remote sensing targets with load attitude information as a known condition [37] will be improved even further. However, our limitation is that we did not take into account the effects of various extreme environments, including environmental changes such as high winds, heavy rain, sand, and dust. We will analyze the stability of our system for UAV payload attitude estimation in different extreme environments in future studies, and we will discuss the effects of different magnetic interference compensation methods on attitude estimation in future studies as well.

## Figures and Tables

**Table 1:** Specific parameters of the MTI-3-8A7G6T module.

Parameter	Reference Value
Gyroscope range	±450°/s
Gyro bias stability	0.003°/s
Roll angle accuracy (static/dynamic)	(0.5°/0.8°)
Pitch angle accuracy (static/dynamic)	(0.5°/0.8°)
Yaw angle accuracy	2°
Accelerometer range	160 m/s^2^
Accelerometer bias stability	0.03 mg
Accelerometer accuracy	0.03 m/s^2^
Magneto metric range	±80 μT

**Table 2:** The key parameters of the experimental algorithm.

Symbol	Meaning	Reference Value
$g_{0}$	Local gravitational acceleration	$9.7944\;m/s^{2}$
m0n	Local geomagnetic field strength	0.52708 Gauss
Δt	The sampling period	0.01 s
$\sigma_{bg}^{2}$	Gyro zero bias	0.003°/s
$\sigma_{a}^{2}$	Accelerometer measurement variance	$0.001\;m/s^{2}$
$\sigma_{m}^{2}$	Magnetometer Noise Variance	0.001 μT
$\sigma_{d}^{2}$	Basic variance of magnetic interference	0.0001 Gauss

**Table 3:** Pose estimation error evaluation in experiment A (RMSE).

Attitude Angle	EKF	ESKF	DEKF	DOEA	HDAEA
Pitch	1.01	0.91	0.43	0.31	0.26
Roll	1.39	1.12	0.57	0.46	0.20
Azimuth	11.41	10.23	6.02	2.79	1.60

**Table 4:** Pose estimation error evaluation in the experiment to verify the validity of the merit function (RMSE).

Attitude Angle	DEKF	DOEA	DEKF + MF	DOEA + MF	HDAEA
Pitch	0.43	0.31	0.43	0.31	0.26
Roll	0.57	0.46	0.57	0.46	0.20
Azimuth	6.02	2.79	4.12	2.13	1.60

**Table 5:** Pose estimation error evaluation in experiment B (RMSE).

Attitude Angle	EKF	ESKF	DEKF	DOEA	HDAEA
Pitch	2.30	1.95	1.38	1.29	0.83
Roll	9.36	4.54	2.65	2.80	1.09
Azimuth	13.98	9.07	6.72	3.61	2.17

## Data Availability

The raw data supporting the conclusions of this article will be made available by the authors upon request.

## Abbreviations

The following abbreviations are used in this manuscript:

HDAEA	Hierarchical decoupled attitude estimation algorithm
UAV	Unmanned aerial vehicle
MEMS	Micro-electro-mechanical systems
AHRS	Attitude and heading reference system
IMU	Inertial measurement unit
KF	Kalman filter
CF	Complementary filter
GDA	Gradient descent algorithm
EKF	Extended Kalman filter
IEKF	Iterated extended Kalman filter
UKF	Unscented Kalman filter
ESKF	Error-state extended Kalman filter
DCM	Direction cosine matrix
3DOF	3 degrees of freedom
MF	Merit function

## References

[1] Aminifar F., Rahmatian F. (2020). Unmanned Aerial Vehicles in Modern Power Systems: Technologies, Use Cases, Outlooks, and Challenges. IEEE Electrif. Mag..

[2] Alhafnawi M., Salameh H.A.B., Masadeh A., Al-Obiedollah H., Ayyash M., El-Khazali R., Elgala H. (2023). A Survey of Indoor and Outdoor UAV-Based Target Tracking Systems: Current Status, Challenges, Technologies, and Future Directions. IEEE Access.

[3] Shakhatreh H., Sawalmeh A.H., Al-Fuqaha A., Dou Z., Almaita E., Khalil I., Othman N.S., Khreishah A., Guizani M. (2019). Unmanned Aerial Vehicles (UAVs): A Survey on Civil Applications and Key Research Challenges. IEEE Access.

[4] Ding W., Jiang Y., Lyu Z., Liu B., Gao Y. (2022). Improved attitude estimation accuracy by data fusion of a MEMS MARG sensor and a low-cost GNSS receiver. Measurement.

[5] Nazarahari M., Rouhani H. (2021). 40 years of sensor fusion for orientation tracking via magnetic and inertial measurement units: Methods, lessons learned, and future challenges. Inf. Fusion.

[6] Bo F., Li J., Wang W., Zhou K. (2023). Robust Attitude and Heading Estimation under Dynamic Motion and Magnetic Disturbance. Micromachines.

[7] Candan B., Soken H.E. (2021). Robust attitude estimation using imu-only measurements. IEEE Trans. Instrum. Meas..

[8] Wei X., Zhang Y., Fan S., Gao W., Shen F., Ming X., Wang Y. (2025). Low Cost IMU Attitude Estimation Algorithm Based on Measurement Adaptive Reduced DCM. IEEE Sens. J..

[9] Nazarahari M., Rouhani H. (2021). Adaptive Gain Regulation of Sensor Fusion Algorithms for Orientation Estimation with Magnetic and Inertial Measurement Units. IEEE Trans. Instrum. Meas..

[10] Lan J., Wang K., Song S., Li K., Liu C., He X., Hou Y., Tang S. (2024). Method for measuring non-stationary motion attitude based on MEMS-IMU array data fusion and adaptive filtering. Meas. Sci. Technol..

[11] Gu H., Jin C., Yuan H., Chen Y., Huang X. (2021). Design and implementation of attitude and heading reference system with extended Kalman filter based on MEMS multi-sensor fusion. Int. J. Uncertain. Fuzziness Knowl.-Based Syst..

[12] Yu Y.-J., Zhang X., Khan M.S.A. (2020). Attitude heading reference algorithm based on transformed cubature Kalman filter. Meas. Control.

[13] Yang C., Ouyang H. (2022). A novel load-dependent sensor placement method for model updating based on time-dependent reliability optimization considering multisource uncertainties. Mech. Syst. Signal Process..

[14] Nazarahari M., Rouhani H. (2021). Sensor fusion algorithms for orientation tracking via magnetic and inertial measurement units: An experimental comparison survey. Inf. Fusion.

[15] Caruso M., Sabatini A.M., Laidig D., Seel T., Knaflitz M., Della Croce U., Cereatti A. (2021). Analysis of the Accuracy of Ten Algorithms for Orientation Estimation Using Inertial and Magnetic Sensing under Optimal Conditions: One Size Does Not Fit All. Sensors.

[16] Kok M., Schon T.B. (2019). A fast and robust algorithm for orientation estimation using inertial sensors. IEEE Signal Process. Lett..

[17] Feng K., Li J., Zhang X., Shen C., Bi Y., Zheng T., Liu J. (2017). A new quaternion-based Kalman filter for real-time attitude estimation using the two-step geometrically-intuitive correction algorithm. Sensors.

[18] Fan B., Li Q., Liu T. (2018). How magnetic disturbance influences the attitude and heading in magnetic and inertial sensor-based orientation estimation. Sensors.

[19] Rong H., Peng C., Chen Y., Lv J., Zou L. (2023). An EKF-based attitude estimator for eliminating the effect of magnetometer measurements on pitch and roll angles. IEEE Trans. Instrum. Meas..

[20] Park S., Park J., Park C.G. (2020). Adaptive attitude estimation for low-cost MEMS IMU using ellipsoidal method. IEEE Trans. Instrum. Meas..

[21] Madgwick S.O.H., Wilson S., Turk R., Burridge J., Kapatos C., Vaidyanathan R. (2020). An extended complementary filter for full-body MARG orientation estimation. IEEE/ASME Trans. Mechatron..

[22] Ding W., Gao Y. A Quaternion Based Error State Kalman Filter for Attitude Estimation Using Low-cost MEMS MARG Sensors. Proceedings of the 2020 IEEE 92nd Vehicular Technology Conference (VTC2020-Fall).

[23] Kok M., Schon T.B. (2016). Magnetometer calibration using inertial sensors. IEEE Sens. J..

[24] Rong H., Peng C., Chen Y., Lv J., Zou L. (2022). A time-efficient complementary Kalman gain filter derived from extended Kalman filter and used for magnetic and inertial measurement units. IEEE Sens. J..

[25] Wohle L., Gebhard M. A robust quaternion based Kalman filter using a gradient descent algorithm for orientation measurement. Proceedings of the 2018 IEEE International Instrumentation and Measurement Technology Conference (I2MTC).

[26] Farahan S.B., Machado J.J.M., de Almeida F.G., Tavares J.M.R.S. (2022). 9-DOF IMU-based attitude and heading estimation using an extended Kalman filter with bias consideration. Sensors.

[27] Bin H., Zhongping F., Yilin X., Meng W., Yu L., Can T., Wei W., Hang X. (2023). Attitude calculation of quadrotor UAV based on extended Kalman filter and complementary filter. J. Phys. Conf. Ser..

[28] Li P., Zhang W.-A., Jin Y., Hu Z., Wang L. (2023). Attitude estimation using iterative indirect Kalman with neural network for inertial sensors. IEEE Trans. Instrum. Meas..

[29] Yang Y., Li F., Gao Y., Mao Y. (2020). Multi-Sensor Combined measurement while drilling based on the improved adaptive fading square root unscented Kalman filter. Sensors.

[30] Yang Y., Liu X., Zhang W., Liu X., Guo Y. (2020). A Fast Weakly-Coupled Double-Layer ESKF Attitude Estimation Algorithm and Application. Electronics.

[31] Xu X., Sun Y., Tian X., Zhou L., Li Y. (2022). A Double-EKF Orientation Estimator Decoupling Magnetometer Effects on Pitch and Roll Angles. IEEE Trans. Ind. Electron..

[32] Sun Y., Xu X., Tian X., Zhou L., Li Y. (2022). A Decoupled Orientation Estimation Approach for Robust Roll and Pitch Measurements in Magnetically Disturbed Environment. IEEE Trans. Instrum. Meas..

[33] Ding L., Wen C. (2024). High-Order Extended Kalman Filter for State Estimation of Nonlinear Systems. Symmetry.

[34] Yi N., Sun W., Zhou X., Chen L., Zhang J., Han D., Sun C. (2023). A Decoupled-DCM Orientation Estimator for Eliminating Influence of Magnetic Interference on Roll Angle and Pitch Angle. IEEE Trans. Instrum. Meas..

[35] Wei X., Fan S., Zhang Y., Gao W., Shen F., Ming X., Yang J. (2025). A robust adaptive error state Kalman filter for MEMS IMU attitude estimation under dynamic acceleration. Measurement.

[36] Chen J., Cui B., Wei X., Zhu Y., Sun Z., Liu Y. (2024). Robust Attitude Estimation for Low-Dynamic Vehicles Based on MEMS-IMU and External Acceleration Compensation. Sensors.

[37] Zhou X., Jia W., He R., Sun W. (2025). High-Precision Localization Tracking and Motion State Estimation of Ground-Based Moving Target Utilizing Unmanned Aerial Vehicle High-Altitude Reconnaissance. Remote Sens..

